# Openings for the Sons of Medical Men

**Published:** 1912-01-27

**Authors:** Richard Arthur

**Affiliations:** President, Immigration League of Australasia.


*To the Editor of* The Hospital.

### OPENINGS FOR THE SONS OF MEDICAL MEN.

*To the Editor of* The Hospital.

Sir,—The sons of medical men in the United Kingdom often enter the profession of which their fathers are members. But I understand that the medical profession is overcrowded in England, and that Mr. Lloyd George is going to make the prospect of entering it more uninviting than ever. Even in Australia, where the conditions at present are somewhat better, the Universities of Sydney, Melbourne, and Adelaide are turning out hundreds of young doctors, and their numbers are increasing faster proportionately than the population. It is a serious question nowadays for a medical man as to whether his son should follow in his footsteps or whether he might not do better by looking elsewhere. After much consideration I decided that my own son should take up farming in Australia, and I am more and more convinced that this decision is in his true interests. He is spending three years at the premier agricultural college in Australia—the Hawkesbury College, near Sydney. There he is learning both the science and the practice of farming, and the fees, including the most excellent board and lodging, are £30 for the first year, £20 for the second, and £10 for the third. Other expenses and pocket-money come to about £20 a year.

At the other colleges in New South Wales £15 is charged for the first year, and the second is free. At the Hawkesbury College the students attend lectures and the laboratory one day, and are in the fields the next, thus obtaining a really practical training. They have plenty of recreation, cricket, tennis, shooting, and swimmimg, with occasional concerts and dances, and they have an alive Students' Christian Union. Now if a lad goes through the medical course in England it means an outlay of about £1,000. For this amount he could obtain not only a thorough training in agriculture in Australia, but could pay a deposit on land and stock it.

The life is undoubtedly a hard and strenuous one, with much monotony, but it is healthy, and the financial rewards are generally ample to a man with grit and perseverance. I recommend the college course so that a lad may be broken in gradually to the life among his fellows, instead of going direct to a farm and facing the continuous toil on it.

There are similar colleges in Victoria and South Australia where young Englishmen are received. A few vacancies at these colleges are reserved for students from abroad, but it would be well for anyone wishing to enter his name to do so as quickly as possible. Our agents in London, at 50 Parliament Street, S.W., would make arrangements to this end if communicated with, or I would be glad to answer any letters if addressed to me at Parliament House, Sydney, N.S.W.—Yours, etc.,

RICHARD ARTHUR, M.D., C.M. Edin.
President, Immigration League of Australasia.

